# Diagnosis of contralateral rare pulmonary cavity metastasis after lung squamous cell carcinoma surgery by electromagnetic navigation: one case report and review of the literature

**DOI:** 10.3389/fmed.2024.1445752

**Published:** 2024-08-19

**Authors:** Zhengjun Li, Xiaoge Wang, Chang Liu, Yi Ren

**Affiliations:** ^1^Department of Thoracic Surgery, Shenyang Chest Hospital, Shenyang, China; ^2^Department of Respiratory Medicine, Shenyang Chest Hospital, Shenyang, China

**Keywords:** electromagnetic navigation, lung cancer associated with cystic airspaces, squamous cell carcinoma, lung cancer, case report

## Abstract

**Background:**

Lung cancer associated with cystic airspaces is a rare disease, and a rare imaging performance of non-small cell lung cancer. Due to the lack of conventional diagnosis methods, it is difficult to rely on imaging diagnosis. Therefore, the definitive diagnosis of these neoplastic lesions remains challenging.

**Case presentation:**

We summarize the follow-up and diagnosis of a rare cystic airspaces lung metastatic carcinoma in an elderly man with annular density shadow in the right inferior lobe 2 years after surgery for squamous cell carcinoma in the left inferior lobe.

**Results:**

During the follow-up of the patient, after the lesion of the lower lobe of the right lung was enlarged, the structural and imaging characteristics were identified, and a special method was selected, namely biopsy of the lesion under the electromagnetic navigation bronchoscope, for clear diagnosis and subsequent treatment.

**Conclusion:**

For pulmonary cystic airspaces, it is important to correctly identify their imaging features. Because of the possibility of malignancy, it is essential to stop the radiological study in time and to acquire the pathological diagnosis by an appropriate method.

## Introduction

Lung cancer associated with cystic air cavity (LCCA) is a rare imaging manifestation, accounting for only 1%–7% of all lung cancers (1). In contrast to the imaging findings of masses or nodules commonly seen in lung cancer, LCCA is characterized primarily by cystic areas (single or multi-cystic) with consolidation and/or ground-glass shadows (2), it is easy to misdiagnose and delay treatment. In total, 80% of the histological types are adenocarcinoma, and the majority are moderately highly differentiated. Squamous cell carcinoma is a relatively rare pathological type (3).

Womack and Graham first suggested in 1941 that pulmonary cystic lesions may be associated with bronchial carcinoma (4), and since then LCCA has been described as a rare disease with the lowest incidence of adenocarcinoma (4, 5). However, pulmonary cavity metastases are rare (6), due to the lack of clear defense against this condition (7). In addition, the number of clinical cases is relatively small, which leads to the risk of early missed diagnosis or misdiagnosis, resulting in delayed treatment (8). Therefore, familiarity with the diagnosis and treatment of this particular type of lung cancer has become an important goal of our future research.

In this case report, we report on a male patient who underwent radical lung cancer surgery 2 years ago for squamous cell carcinoma in the left inferior lobe of the lung. A rare radiographic first appearance of an empty metastatic lung squamous cell carcinoma in the right inferior lobe of the lung was found in the postoperative follow-up. The focus of the review will be on the imaging changes following the postoperative examination, as well as the diagnosis under electromagnetic navigation, including treatment.

## Case presentation

A 58-year-old male patient, who had smoked 20 cigarettes a day for 40 years, was admitted to the respiratory department with cough and fever for 1 week. Computed tomography (CT) examination was performed at the district hospital to consider the possibility of left low lobe pneumonia. He had no underlying comorbidities. The patient had no previous history. At the time of initial medical examination, blood pressure (BP) was 122/72 mmHg and pulse was 89 beats per minute (bpm). Heart sound is normal, lungs clear, no dry or wet rales auscultation.

Routine laboratory tests were normal, including complete blood count, serum urea and electrolyte levels. Tumor markers [carcinoembryonic antigen (CEA), cytokeratin 19 fragment (CYFRA), pro-gastrin-releasing peptide (ProGRP), neuron-specific enolase (NSE), and squamous cell carcinoma antigen (SCC-Ag)] were in the normal range. After normal body temperature was treated with antibiotics, enhanced chest CT showed stenosis of the lumen at the base of the left inferior lobe with obstructive pneumonia. The mass was about 4 cm in size.

**FIGURE 1:**
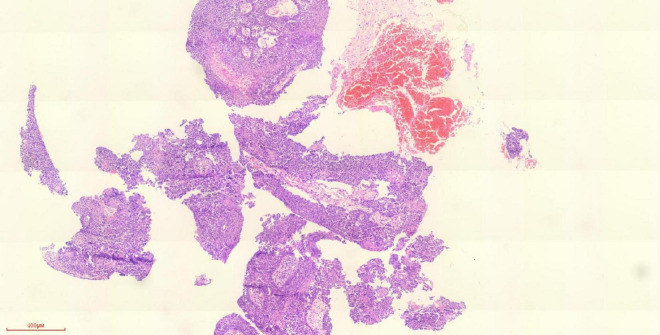
Pathology of tracheoscopy biopsy of left lower lobe of lung (H&E staining) (the lesions of the left lower lobe were found, and the pathology was obtained by tracheoscopy).

Bronchoscopy revealed a new organism in the basal segment of the left lower lobe, the imaging findings were mainly obstructive pneumonia, with no cystic changes (Supplementary Figure 1). And pathological biopsy diagnosed it as lung squamous cell carcinoma (Figure 1), with clinical stage cT2N1M0, no positron emission tomography-computed tomography (PET-CT) examination and invasive mediastinal evaluation were performed before surgery. After completing the examination of cardiopulmonary function, the left lower lobe of lung was excised and the mediastinal lymph node dissection was performed. The postoperative pathology was squamous cell carcinoma without lymph node metastasis. According to the TNM stage of the eighth edition of lung cancer, the pathological stage was pT2bN0M0, stage IIA. After four cycles of gemcitabine (GEM) plus cisplatin (DDP) (GP) regimen chemotherapy was given, regular review was conducted.

**FIGURE 2:**
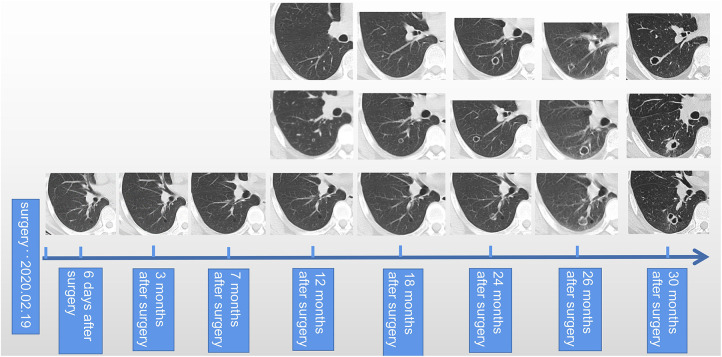
Summary of longitudinal changes in imaging.

The patient’s imaging data were reviewed (Figure 2). One year after surgery, the patient’s chest CT showed a circular high-density shadow in the lower lobe of the right lung, with a size of about 1 mm. The patient was asymptomatic, and the tumor markers were normal. One and a half years after surgery, chest CT showed that the circular high-density shadow in the lower lobe of the right lung was slightly larger than before, about 4 mm, and a similar small circular high-density shadow appeared below, about 1 mm in size. At this time, the patient still had no symptoms, and the tumor markers were normal. No special treatment was performed. Two and a half years after the operation, the two annular high-density shadows increased, forming cavity lesions.

**FIGURE 3:**
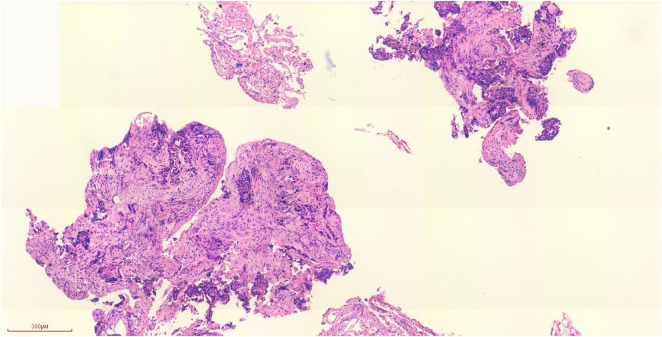
Pathological biopsy of right lower lobe by electromagnetic navigation (H&E staining).

Multidisciplinary consultation (MDT) was performed to determine the diagnosis, excluding the possibility of specific infections (including fungi, cryptococcus), immune-related lung disease, and vasculitis. However, both the interventional department and endoscopy department were informed that neither percutaneous puncture biopsy nor bronchoscopy could obtain histopathology. Considering the previous left inferior lobe surgery and lung function and uncertainty about the effect of surgery, surgical treatment should be carried out cautiously. Moreover, percutaneous biopsy is difficult because the lesion is cystic and the capsule wall is very thin, and the lesion is located at the edge of the subsegmental bronchus. So we adopted the method of bronchoscopic biopsy under electromagnetic navigation to diagnose squamous cell carcinoma of the right inferior lobe of the lung (Figure 3 and Supplementary Figure 2).

Immunohistochemical diagnosis and gene detection were performed on both tissues. The gene detection of 2020s surgical specimens showed TP53 mutation, epidermal growth factor receptor (EGFR) amplification, CCND1 amplification and PIK3CA amplification; in 2022, the gene detection of pathological specimens under magnetic navigation showed TP53 mutation, CDKN2A mutation, CCND1 amplification, and PIK3CA amplification. There is no gold standard to diagnose whether the two are homologous. According to literature reports, the two can be considered homologous if they share a common driver mutation or the mutation agreement rate is greater than or equal to 90%. Combined with histological and molecular evaluation, the two lesions were homologous, so we determined that the lesion in the lower lobe of the right lung was metastatic lung squamous cell carcinoma. Albumin binding paclitaxel + cisplatin chemotherapy was followed, and the efficacy was evaluated partial response (PR). At the time of the draft, the patient received the second cycle of chemotherapy. The patient was aware of the imaging and treatment difficulties and was very satisfied with the whole treatment process (Supplementary Figure 3). After two cycles of chemotherapy, the patient refused further chemotherapy, and after 1 month imaging evaluation was PR. Later the patient was lost to follow-up.

## Discussion

Lung cancer associated with cystic air gap is still considered a relatively rare tumor, and its imaging findings are rare in non-small cell lung cancer (NSCLC). Only recently 10 years attention was drawn to a possible association between lung cancer and smaller cystic airspaces (9). In Fintelmann et al. (10) showed that LCCA accounts for about 1% of NSCLC, most of which are classified as adenocarcinoma, squamous cell carcinoma is relatively rare, and is found in former and current smokers with emphysema. Hollow lung metastatic malignant lesions are more rare, accounting for about 4% of metastatic lesions (11). Vacuous metastasis of lung is more common in squamous cell carcinoma of head and neck, gastrointestinal tract, adenocarcinoma of breast, prostate cancer, sarcoma, etc. (12–15). Vacuous metastasis of lung squamous cell carcinoma is extremely rare.

Both primary and metastatic lesions have a low overall prevalence and are easily missed by radiologists and respiratory doctors (16). Early diagnosis can be challenging and may be advanced by the time it is detected.

Early cystic lung cancer may only show thin-walled cystic structures, and it is easy to be misdiagnosed as pulmonary bulla and pulmonary cyst. Therefore, planned observation and diagnosis are needed. Therefore, CT image features and possible pathogenesis may help us to understand and diagnose these diseases (10, 17). In the current radiological system that classifies all LCCAs, Mascalchi et al. (5, 18) have described four morphological types of external cystic carcinoma: Type I represents nodules outside the cystic airspace and adjacent to the cell wall. Type II is a nodule protruding from the wall into the cystic airspace. Type III is a thickened cyst wall, not necessarily circular, with no area of focal nodules. Type IV is a polycystic disease with a focal soft tissue component. At present, only a few studies have retrospectively analyzed the imaging diagnostic criteria or the longitudinal management of these diseases (9, 19). The case described here is an intrapulmonary metastatic tumor of lung squamous cell carcinoma, with a progression similar to that of type III. In addition, Jung et al. (20) elaborated the imaging morphological changes of LCCA in different stages of development, and reproduced the natural clinical course and clinically related pathological features of LCCA. The true pathogenesis of cystic airspace has not been fully understood. Several different causes have been described (21–25), including (1) central tumor necrosis; (2) the valve mechanism of small airway dilation; (3) direct destruction of lung cells; (4) the squamous growth of essentially adenocarcinoma of the lung in emphysema; (5) cancers caused by clusters of intramural mucous cells in such congenital pulmonary airway malformations; (6) the adenocarcinoma grows along the wall of the existing bullae; and (7) autophagy of cancer cells. We believe that there may be a combination of mechanisms leading to the development of these lesions.

We report a case of lung squamous cell carcinoma with pulmonary cavity metastasis. With the progression of the lesion, progressive thickening of the cyst wall or the appearance or enlargement of nodules adjacent to the cystic area may occur, and the diameter of the cystic airspace may decrease, increase or remain stable (7), and part of it may become solid (18). If the progression of such diseases is fully understood from imaging, early diagnosis and treatment may change the prognosis (26). Fintelmann et al. (10) reported that the median time between the observation of similar imaging abnormalities and the diagnosis of lung cancer was 25.5 months.

Positron emission tomography-computed tomography has many shortcomings in the diagnosis of such diseases. The gas-containing characteristics of the lesion morphology can reduce the total density of metabolically active cells and reduce the uptake of fuorine-18-fuorodeoxyglucose (FDG). Negative PET results cannot reliably exclude malignant tumors (3, 17, 18, 27). The lesion in the case we described had a lower uptake than the bronchial stump of the left inferior lobe that had been defined. Mascalchi et al. (5, 18) reported 24 cases of lung cancer with cystic air gaps. The diagnosis of malignancy was based on cytological examination by CT-guided fine-needle aspiration biopsy (FNAB) (=18) or by surgical specimen or core biopsy (=6) (7). Mendoza et al. (19) found that more than 300 cases of LCCA were reported in the form of case reports or small case series, most of which were diagnosed through surgery, which was consistent with the report of Farooqi et al. (9) and Guo et al. (28). In clinical practice, transbronchial biopsies and percutaneous lung biopsies often end in failure, so we should be aware of the limitations of preoperative biopsies in diagnosing these patients. Thoracoscopic surgery is essential as a minimally invasive operation for patients with highly suspected lung cancer. Therefore, it is our opinion that CT follow-up of cavernous lesions should be terminated in time, and the best means such as electromagnetic navigation bronchoscopic biopsy should be used to obtain pathological diagnosis for timely follow-up treatment.

The majority of cancers associated with cystic airspaces are of the adenocarcinoma type, squamous cell carcinoma is relatively rare, while lung squamous cell carcinoma metastatic cavity changes are rare. By evaluating the imaging features of the lesions, we selected the electromagnetic navigation bronchoscopy technique with a higher success rate of tissue acquisition, and made pathological diagnosis. It provides great help for the follow-up and timely treatment. Compared with the primary disease, it is more important to distinguish the primary disease from the metastatic disease in this case, which was evaluated by comprehensive histologic assessment (CHA) (29) and molecular evaluation (30). Histologically, including immunohistochemistry, most of the morphology and expression were consistent. As reported in the literature, if the molecular expression of the two lesions of solid tumor showed high consistency, it could be considered homology (31), that is, considering the metastasis. In our case, it is important to stop the observation in time and obtain the pathology by appropriate methods, because percutaneous biopsy and PET-CT are very limited. Electromagnetic navigation bronchoscopy biopsy is more advantageous, and even active surgery can be considered. And the further mutation of the two genetic tests was 75% consistent, and the two lesions shared a TP53 mutation, so we determined the lesion on the right as metastatic tumor. We believe that electromagnetic navigation bronchoscopy has less limitations than conventional biopsy and surgery, especially in such patients, cystic lesions are mostly near the subsegmental bronchus, with better indications and positive rates.

## Conclusion

Therefore, it is important to recognize the imaging features and longitudinal progression of these diseases, both primary and metastatic, and to recognize the limitations of preoperative biopsy and PET-CT. Timely and effective pathological diagnosis (such as surgery and electromagnetic navigation) is essential for diagnosis and treatment.

## Data Availability

The original contributions presented in this study are included in this article/Supplementary material, further inquiries can be directed to the corresponding author.

## Supplementary material

Supplementary Figure 1 Preoperative chest CT.

Supplementary Figure 2 Electromagnetic navigation bronchoscopic biopsy.

Supplementary Figure 3 Timeline figure of case treatments.
