# Supplementary material for: Diagnosis of contralateral rare pulmonary cavity metastasis after lung squamous cell carcinoma surgery by electromagnetic navigation: one case report and review of the literature
Source: Front Med (Lausanne). 2024 Aug 19;11:1445752. doi: 10.3389/fmed.2024.1445752 (PMC11375509; doi:10.3389/fmed.2024.1445752)
Supplement: Supplementary file 1 [file Table_1.pdf]

CARE case report guidelines

CARE Checklist of information to include when writing a case report

(cc) BY-NC-ND

Topic	Item	Checklist item description	Reported on Line
Title	1	The diagnosis or intervention of primary focus followed by the words "case report"	2
Key Words	2	2 to 5 key words that identify diagnoses or interventions in this case report, including "case report"	20-21
Abstract (no references)	3a	Introduction: What is unique about this case and what does it add to the scientific literature?	44-46
	3b	Main symptoms and/or important clinical findings	77-78
	3c	The main diagnoses, therapeutic interventions, and outcomes	84-101
	3d	Conclusion—What is the main "take-away" lesson(s) from this case?	43-46
Introduction	4	One or two paragraphs summarizing why this case is unique (**may include references**)	112-133
Patient Information	5a	De-identified patient specific information.	78-83
	5b	Primary concerns and symptoms of the patient.	84-101
	5c	Medical, family, and psycho-social history including relevant genetic information	78-83
	5d	Relevant past interventions with outcomes	91-98
Clinical Findings	6	Describe significant physical examination (PE) and important clinical findings.	102
Timeline	7	Historical and current information from this episode of care organized as a timeline	102、137
Diagnostic Assessment	8a	Diagnostic testing (such as PE, laboratory testing, imaging, surveys).	84-95
	8b	Diagnostic challenges (such as access to testing, financial, or cultural)	105-111
	8c	Diagnosis (including other diagnoses considered)	118-122
	8d	Prognosis (such as staging in oncology) where applicable	133-139
Therapeutic Intervention	9a	Types of therapeutic intervention (such as pharmacologic, surgical, preventive, self-care)	120-122
	9b	Administration of therapeutic intervention (such as dosage, strength, duration)	133-134
	9c	Changes in therapeutic intervention (with rationale)	118-134
Follow-up and Outcomes	10a	Clinician and patient-assessed outcomes (if available)	133-134
	10b	Important follow-up diagnostic and other test results	135-139
	10c	Intervention adherence and tolerability (How was this assessed?)	118-120
	10d	Adverse and unanticipated events	125-131
Discussion	11a	A scientific discussion of the strengths AND limitations associated with this case report	158-160
	11b	Discussion of the relevant medical literature **with references**.	143-200
	11c	The scientific rationale for any conclusions (including assessment of possible causes)	182-189
	11d	The primary "take-away" lessons of this case report (without references) in a one paragraph conclusion	231-235
Patient Perspective	12	The patient should share their perspective in one to two paragraphs on the treatment(s) they received	135-139
Informed Consent	13	Did the patient give informed consent? Please provide if requested	Yes ☑ No ☐
