# Supplementary figures and images for: Diagnosis of contralateral rare pulmonary cavity metastasis after lung squamous cell carcinoma surgery by electromagnetic navigation: one case report and review of the literature
Source: Front Med (Lausanne). 2024 Aug 19;11:1445752. doi: 10.3389/fmed.2024.1445752 (PMC11375509; doi:10.3389/fmed.2024.1445752)

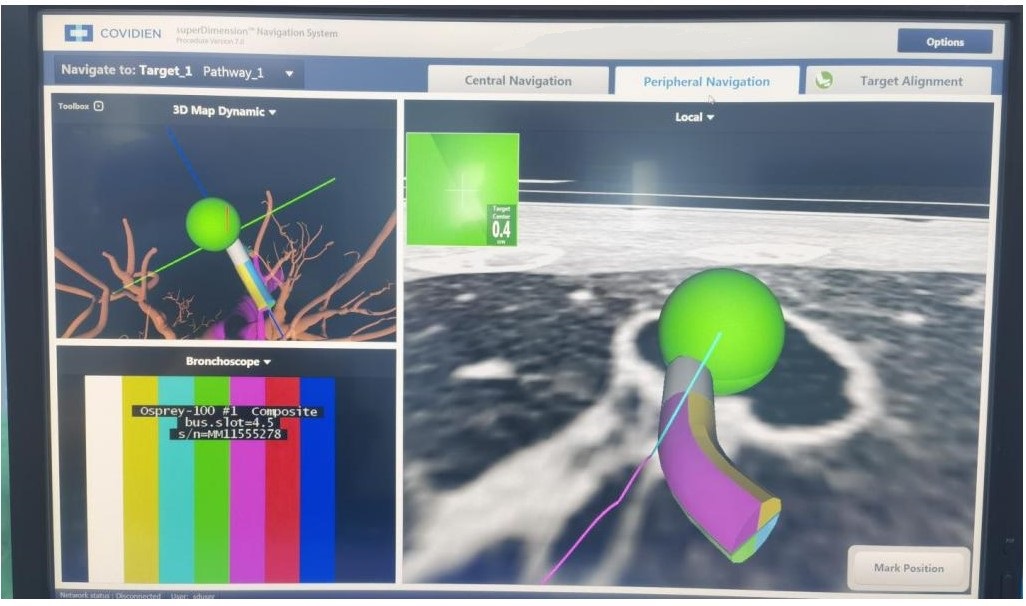

Supplement: Supplementary Figure 1 — Preoperative chest CT. [file Image_1.jpg]

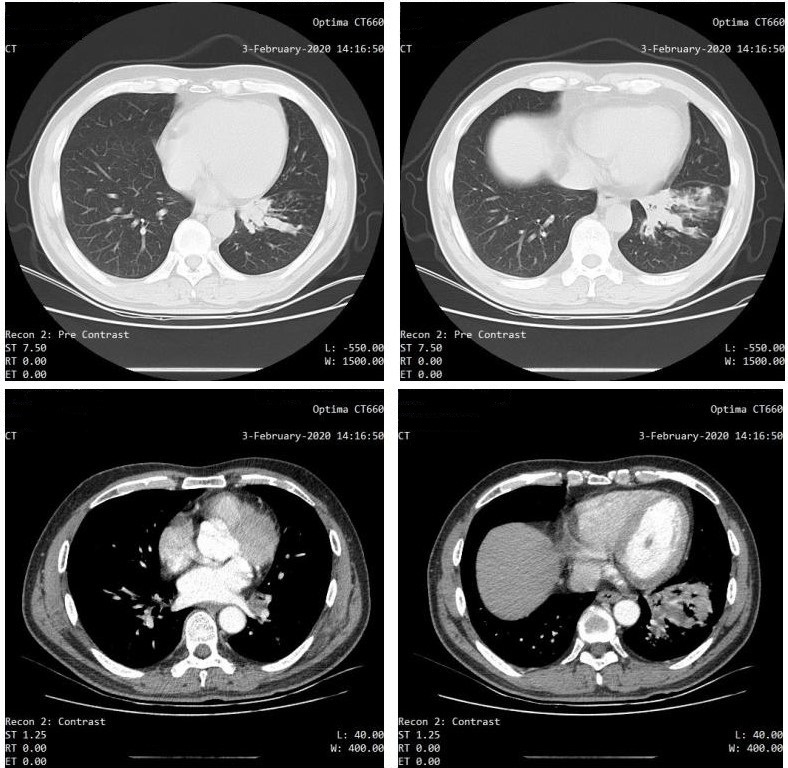

Supplement: Supplementary Figure 2 — Electromagnetic navigation bronchoscopic biopsy. [file Image_2.jpg]

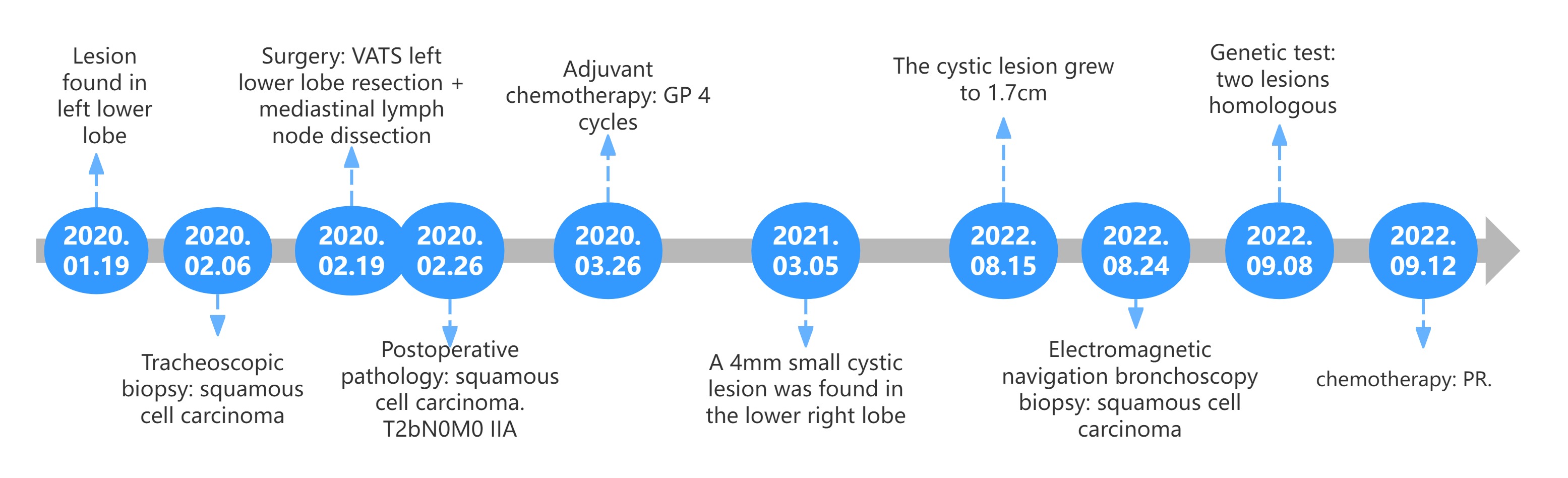

Supplement: Supplementary Figure 3 — Timeline figure of case treatments. [file Image_3.JPEG]
